# A model based on endorectal ultrasonography predicts lateral lymph node metastasis in low and middle rectal cancer

**DOI:** 10.1002/jcu.23204

**Published:** 2022-03-24

**Authors:** Li Yan, Zhou Weifeng, Wang Qin, Wang Jinping

**Affiliations:** ^1^ Department of Ultrasound The first affiliated hospital of Anhui University of Traditional Chinese Medicine Anhui China

**Keywords:** ERUS, lymph node metastasis, rectal cancer, SR

## Abstract

**Purpose:**

To investigate the risk factors for lymph node (LN) metastasis in low and middle rectal tumors, construct a predictive model and test the model's diagnostic efficacy.

**Methods:**

The clinical and pathological data of 172 patients with rectal cancer confirmed by surgery were retrospectively evaluated, among whom 61 patients were finally included in this study. Patients were divided into positive groups and negative groups based on LN metastasis, and risk factors that might affect LN metastasis were analyzed. Finally, a risk predictive model was constructed based on the weights of each risk factor.

**Results:**

Compared with pathology, the efficacy of diagnosing LN metastasis only according to conventional endorectal ultrasonography (ERUS) features of LN was not high, with sensitivity 67%, specificity 86%, positive predictive value 76%, negative predictive value 80%, and accuracy 79%. Univariate analysis showed that circumferential angle of the tumor, ultrasonic T‐ stage (UT stage), conventional ultrasound features diagnosis of LN metastasis, strain ratio (SR) of tumor were risk factors for LN metastasis, while vascular resistance index of rectal tumor was protective factor. Multivariate analysis showed that UT stage (OR = 7.188, *p* = 0.049), conventional ultrasound features diagnosis of LN metastasis (OR = 8.010, *p* = 0.025) and SR (OR = 5.022, *p* = 0.031) were independent risk factors for LN metastasis. These risk factors were included in logistic regression analysis and the model was established, *Y* = −7.3 + 1.9 X10 + 2.1 X11 + 1.6 X13 (*Y* = Logit[*P*], *P*: LN metastasis rate, X10: UT stage, X11: conventional ultrasound features diagnosis of LN metastasis, X13: SR). The receiver operating characteristic (ROC) curve was used to test the model's predictive efficacy, the area under the curve was 0.95, sensitivity: 95%, specificity: 87%. Hosmer–Lemeshow goodness of fit test showed X2 = 6.015, *p* = 0.65 (*p* > 0.05), indicating that the model had a high predictive value.

**Conclusion:**

Evaluation of perirectal LN metastasis only based on conventional ERUS features of LN was not effective enough. UT stage of tumor, conventional ultrasound features diagnosis of LN metastasis and SR were independent risk factors for LN metastasis. The predictive model had good assessment efficacy and had certain clinical application value.

## Graphical abstract

Multivariate analyses showed the independent risk factors for LN metastasis was UT stage, conventional ultrasound features diagnosis of LN metastasis and SR. These independent risk factors were included in logistic regression analysis and the model was established, *Y* = −7.3 + 1.9X10 + 2.1X11 + 1.6X13 (*Y* = Logit [*P*], *P*: LN metastasis rate, X10: UT stage, X11: conventional ultrasound features diagnosis of LN metastasis, X13: SR). The ROC curve was used to test conventional ultrasound and the model's predictive efficacy, the area under the curve was 0.76 (95% CI: 64%–89%, *p* = 0.000), 0.95 (95% CI: 90%–99%, *p* = 0.000), respectively. Hosmer–Lemeshow goodness of fit test indicated this model had a high predictive value. The cutoff of the model was −0.80.

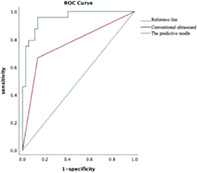

## 1 INTRODUCTION

Currently, rectal carcinoma is one of the common gastrointestinal tumors and the incidence is increasing.^1^, ^2^ Lymph node (LN) metastasis is the main way of rectal cancer metastasis. Mesenteric LN is the most frequently involved tissue of rectal cancer diffusion and metastasis.^3^ For benign tumors and early rectal cancer without LN metastasis, local resections such as endoscopic dissection and transanal endoscopic minimally invasive surgery can be selected to achieve a radical cure.^4^ Such treatments reduce the damage to the patient and economic burden and improve the quality of life. If perirectal LNs metastasize, the draining regional LNs are likely to have metastasized, which is an indication of neoadjuvant chemoradiotherapy.^5^ If minimally invasive surgery is selected blindly, treatment will be delayed. Therefore, accurate preoperative assessment of LN metastasis is critical to the selection of appropriate treatment, as both inadequate and excessive treatment will have a significant impact on the prognosis of patients.

Evaluation of LN metastases becomes critical in developing a treatment plan. Unfortunately, there are no clinical findings or imaging examinations that are very accurate in assessing LN metastases before surgery. Endorectal ultrasonography (ERUS) and MRI are the preferred examinations for rectal tumors. Previously, these tests were used to determine whether a LN was metastatic by morphologic characterization of the LN, however the accuracy was not high.^6^, ^7^ In addition, it cannot meet the requirements of precision medicine only by one index.^8^ In this study, multiple factors related to LN metastasis were incorporated into Logistic regression to analyze the independent influencing factors of perirectal LNs metastasis.

## 2 MATERIALS AND METHODS

### 2.1 Patients

**FIGURE 1:**
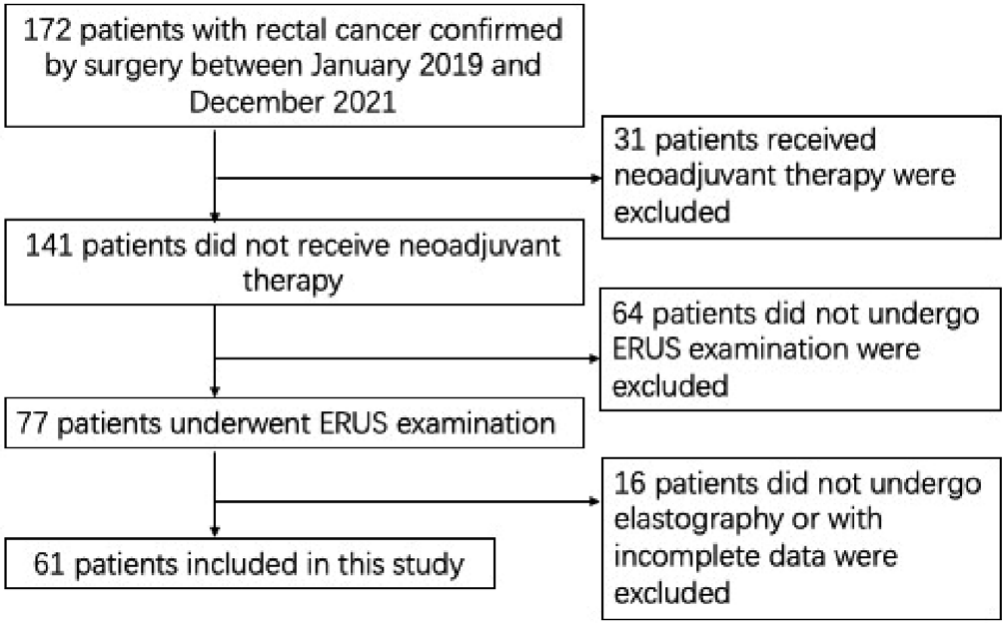
Workflow of patient selection

This study was approved by the Hospital Ethics Council (number: 2021MCZQ12). From January 2019 to December 2021, 172 patients confirmed rectal cancer pathologically were recruited into this study. Patients received neoadjuvant therapy, did not undergo ERUS examination or with incomplete data were excluded, 61 patients were finally included in this study, including 31 males and 30 females, with an age range of 33–86 years. The workflow of screening patients is shown in Figure 1.

Inclusion criteria: (1) ERUS was performed within 2 weeks before surgery; (2) rectal tumor within 10 cm from the anal margin; (3) No neoadjuvant chemoradiotherapy was performed before examination; (4) Histopathological diagnosis was performed postoperatively.

Exclusion criteria: (1) incomplete ERUS results due to intestinal stenosis or high tumor location; (2) recurrent rectal cancer; (3) complicated with other types of malignant tumors; (4) the patient had a history of pelvic infection; (5) pregnancy.

### 2.2 Image acquisition

#### 2.2.1 Instrument

HITACHI ARIETTA 70 ultrasonic instrument was used, with a 360° transrectal probe and a biplane probe, frequency: 5–10 MHz, the frequency could be adjusted. In addition, one‐time retention enema bags and physiological saline were prepared.

#### 2.2.2 Procedure

##### Preparation

(1) Applied the couplant to the surface of the rectal ultrasound probe and covered it with a clean condom. (2) In order to get the patient's cooperation, before the examination, the sonographers explained to the patient about the purpose of the examination to eliminate the patient's tension. (3) Two days before the examination, patients should eat less residue diet, one day before, take some liquid food, before the examination, warm water enema one to two times to remove the residue in the rectum. At the time of examination, 100–200 mL of normal saline was kept in the rectum.

##### Detection techniques

The patient was placed in the comfortable left decubitus position and the anus was exposed. First, the examiner performed an anal finger examination to see whether there was lumen stenosis, then the probe was slowly inserted into the anus, and the patient was instructed to relax the abdomen and anus during the insertion. After the probe entered the anal canal, the rectum was scanned layer by layer. After the routine ERUS examination, we switched to real‐time elastography mode. The patient was instructed to do Valsalva action to pressurize the probe, adjusted the force according to the displayed pressure index, controlling the pressure between 2 and 3, stored the stable elastic image for 30–60 s, and measured the strain ratio (SR) value of the tumor through the software that came with the instrument, measured three times in a row and took the average value. The diagnosis of each patient was completed by the same two doctors, who had 5 years of experience in ERUS. The operator was unaware of pathological diagnosis, laboratory examination results, and other imaging examination results, but knew the patient's clinical symptoms. The intraclass correlation coefficient was used to evaluate the reproducibility of the examination results of the two sonographers.

### 2.3 Observed indicator

#### 2.3.1 Ultrasonic observation index

**FIGURE 2:**
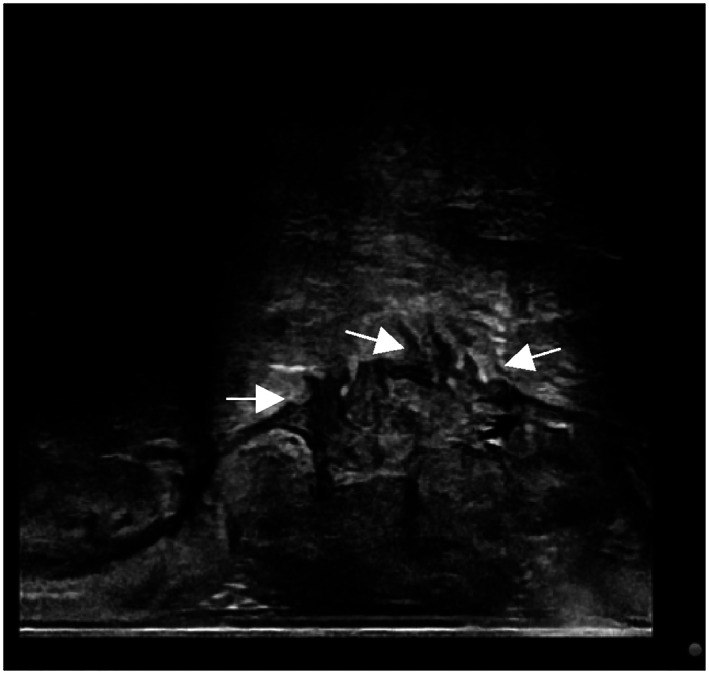
ERUS high‐frequency probe showed tumor invasion into the fatty layer outside the intestinal wall

1. Ultrasonic T‐ stage (UT stage) (Figure 2)

The diagnosis of UT stage was based on Beynon's staging criteria.^9^

**FIGURE 3:**
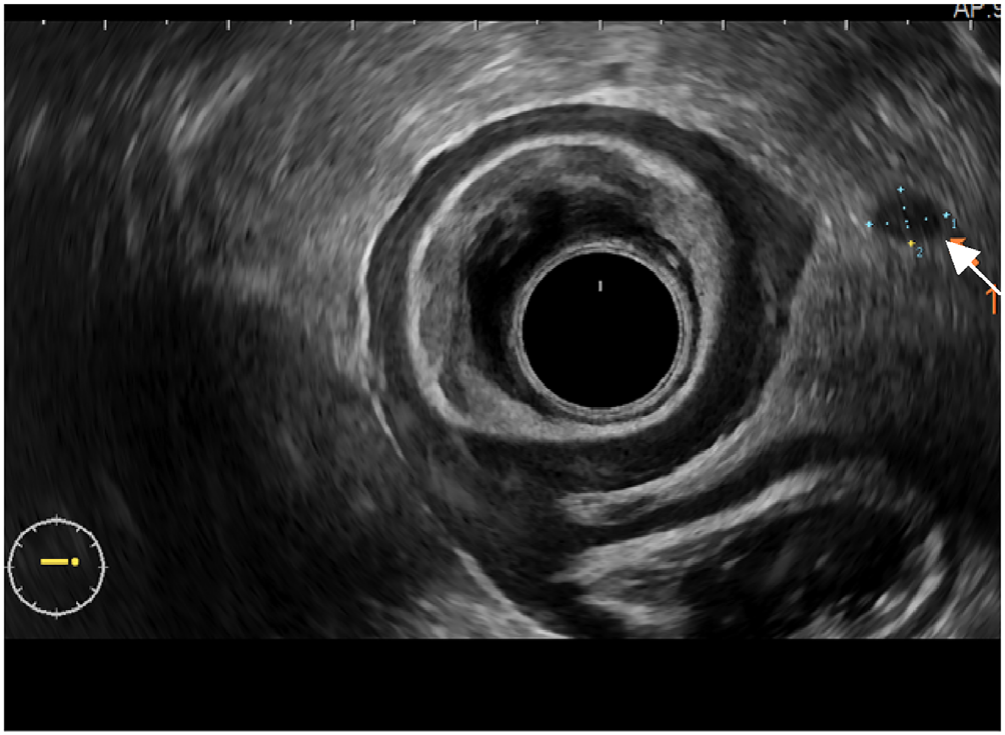
ERUS 360° probe showed abnormal lymph nodes (oval, hypoechoic, long size:6 mm)

**FIGURE 4:**
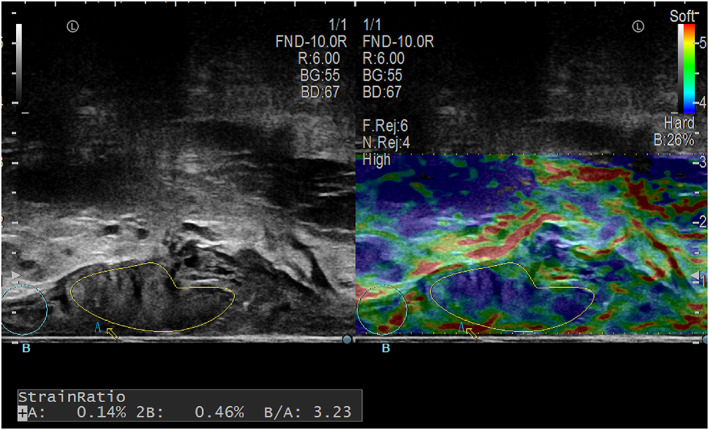
Image shows ERUS B‐Mode on the left and elastography on the right. SR = *B*/*A*, *A* is the region of tumor, *B* is the region of the normal intestinal wall

2. Diagnosis of perirectal LNs metastasis by conventional ultrasound morphological features^9^, ^10^ (Figure 3): LNs that are round and hypoechoic and >5 mm in diameter tend to be considered metastatic.
3. Other indicators recorded are tumor orientation, size, morphology, tumor distance from anal margin, circumferential angle of the tumor, peak systolic velocity (PSV), vascular resistance index (RI), SR (SR = strain rate of normal intestinal wall/strain rate of lesion)^10^ (Figure 4).

#### 2.3.2 Clinical indicators

Gender, age, carbohydrate antigen 19‐9 (CA19‐9), carcinoembryonic antigen (CEA), and other related data. Pathological results of LNs: the patient received surgical treatment within 2 weeks after the ultrasound examination, and the specimen was taken during the operation and sent for pathology examination quickly.

### 2.4 Statistical methods

SPSS 26.0 statistical software was used for analysis. The measurement data were tested for normality first, and the *T* test was used for comparison between the two groups for data conforming to normal distribution, while the Mann–Whitney *U* test was used for nonnormally distributed data. Spearman correlation was used for correlation analysis. *X* ^2^ test was used for comparison of counting data. The diagnostic accuracy was calculated using surgical pathology as the gold standard. The variables with differences in univariate analysis were incorporated into Logistic regression analysis and the diagnostic model was established. Receiver operating characteristic (ROC) curve was used to evaluate the diagnostic efficiency of model. *p* < 0.05 was considered statistically significant.

## 3 RESULTS

### 3.1 The participant characteristics are shown in Table 1

**TABLE 1:** Characteristics of the participant

Characteristics	n = 61
Age	64 (51, 73)
Gender, no (%)	
Male	31 (50.8%)
Female	30 (49.2%)
CA19‐9 (U/mL)	5.57 (2.94, 11.7)
CEA (ng/mL)	3.54 (1.79, 4.96)
Tumor distance from anal margin (cm)	5 (4.5, 7)
Long size of tumor (cm)	2.9 (2.2, 3.9)
RI	0.67 ± 0.06
PSV (cm/s)	18.3 ± 1.41
SR	2.53 (2.29, 3.35)
UT‐stage	
UT0–1	31 (50.8%)
UT2	17 (27.8%)
UT3	13 (21.3%)
Circumferential angle of the tumor	
≤1/2	30 (49.2%)
>1/2	31 (50.8%)
Tumor morphology	
Protruded	29 (47.5%)
Flat or concave	32 (52.5%)

*Note*: Nonnormally distributed variables were presented as median (IQR), and x ± s for normally distributed variables.

### 3.2 Table 2 was the list of assignment description, and some continuous variables were converted into classified variables

**TABLE 2:** Assign values to variables

Variate		Valuation
X1	Age	≤60 = 0, >60 = 1
X2	Gender	Male = 0, Female = 1
X3	CA19‐9 (U/mL)	≤37 = 0, >37 = 1
X4	CEA(ng/mL)	≤5 = 0, >5 = 1
X5	Tumor distance from anal margin	<5 cm = 0, 5–10 cm = 1
X6	Circumferential angle of the tumor	≤1/2 = 0, >1/2 = 1
X7	Tumor morphology	Protruded = 0, Flat or concave = 1
X8	Long size of tumor	≤3.5 cm = 0, >3.5 cm = 1
X9	RI	≤0.70 = 0, >0.70 = 1
X10	UT stage	T0–1 = 0, T2–3 = 1
X11	Conventional ultrasound features diagnosis of LN metastasis	No = 0, Yes = 1
X12	PSV (cm/s)	Continuous variable
X13	SR	Continuous variable

### 3.3 Comparison between conventional ERUS ultrasound feature evaluating lymph node metastasis and pathology

Table 3 showed the comparison between pathology results and conventional ERUS ultrasound feature evaluating LN metastasis. The results showed evaluation of perirectal LN metastasis only based on conventional ERUS features was not effective enough. The correlation between separate conventional ERUS ultrasound features and LN metastasis was weak, *r* = 0.54, *p* = 0.000.

**TABLE 3:** Comparison between conventional ERUS ultrasound feature evaluating lymph node metastasis and pathology

Ultrasound	Pathology							
	−	+		Accuracy	SE	SP	PPV	NPV
−	32	8	40	79%	67%	86%	76%	80%
+	5	16	21					
	37	24	61					

Abbreviations: NPV, negative predictive value; PPV, positive predictive value; SE, sensitivity; SP, specificity.

### 3.4 Comparison of clinical and ultrasonic characteristics between positive group and negative group of lymph node metastases

Table 4 showed the results of univariate analyses. Circumferential angle of the tumor, UT stage, conventional ultrasound features diagnosis of LN metastasis, SR of tumor were risk factors for LN metastasis, while RI of rectal tumor was protective factor, the difference was statistically significant (*p* < 0.05). Other factors (age, gender, CA19‐9, CEA, distance from anal margin, morphology, long size of tumor, PSV) had no significant effect on LN metastasis.

**TABLE 4:** Comparison of clinical and ultrasonic characteristics between positive group and negative group of lymph node metastases

Factors		Lymph node (−) (n = 37)	Lymph node (+) (n = 24)	*t*/*z*/*X* ^2^	*p*
X1	Age				
	≤60	13	9	0.035	0.851
	>60	24	15		
X2	Gender				
	Male	20	11	0.394	0.530
	Female	17	13		
X3	CA19‐9 (U/mL)				
	≤37	31	18	0.711	0.399
	>37	6	6		
X4	CEA (ng/mL)				
	≤5	25	14	0.538	0.463
	>5	12	10		
X5	Tumor distance from anal margin				
	<5 cm	14	5	1.963	0.161
	5–10 cm	23	19		
X6	Circumferential angle of the tumor				
	≤1/2	22	8	3.967	0.046
	>1/2	15	16		
X7	Tumor morphology				
	Protruded	21	8	3.203	0.074
	Flat or concave	16	16		
X8	Long size of tumor(cm)				
	≤3.5	25	13	1.113	0.291
	>3.5	12	11		
X9	RI				
	≤0.70	8	20	25.333	<0.001
	>0.70	29	4		
X10	UT stage				
	T0–1	27	4	18.466	<0.001
	T2–3	10	20		
X11	Conventional ultrasound features diagnosis of LN metastasis				
	N (−)	32	8	18.219	<0.001
	N (+)	5	16		
X12	PSV (cm/s)	17.98 ± 1.19	18.51 ± 1.38	1.592	0.117
X13	SR	2.291 (1.49)	3.33 (0.40)	4.930	<0.001

### 3.5 Multivariate analyses

Table 5 showed the results of multivariate analyses: UT stage, conventional ultrasound features diagnosis of LN metastasis and SR were independent risk factors for LN metastasis. Rectal tumor with T2–3 had a significantly higher risk of LN metastasis than T0–1 (OR = 7.188, *p* = 0.049). The risk of LN metastasis was increased if LNs were assessed as metastatic by conventional ultrasound features (OR = 8.010, *p* = 0.025). For every unit increase in SR, the risk of LN metastasis from rectal cancer increased fivefold approximately (OR = 5.022, *p* = 0.031).

**TABLE 5:** Multivariate analyses of lymph node metastasis

Factors	β	OR	95% CI	*p*
X6	1.417	4.125	0.678–25.101	0.124
X9	−1.761	0.172	0.026–1.131	0.067
X10	1.972	7.188	1.002–51.572	0.049
X11	2.081	8.010	1.306–49.114	0.025
X13	1.614	5.022	1.159–21.767	0.031
Intercept	−7.339	–	–	0.010

Abbreviations: X6, circumferential angle of the tumor; X9, RI; X10, UT stage; X11, conventional ultrasound features diagnosis of LN metastasis; X13, SR.

### 3.6 Construction of Logistic regression model for risk of lymph node metastasis

These independent risk factors were included in logistic regression analysis and the model was established, *Y* = −7.3 + 1.9X10 + 2.1X11 + 1.6X13 (*Y* = Logit [*P*], *P*: LN metastasis rate, X10: UT stage, X11: conventional ultrasound features diagnosis of LN metastasis, X13: SR). The −2logarithm likelihood ratio of the model was 34.336, Cox & Snell *R* Square = 0.54, N Agelkerke *R* Square = 0.73.

### 3.7 The predictive efficacy of the model

**FIGURE 5:**
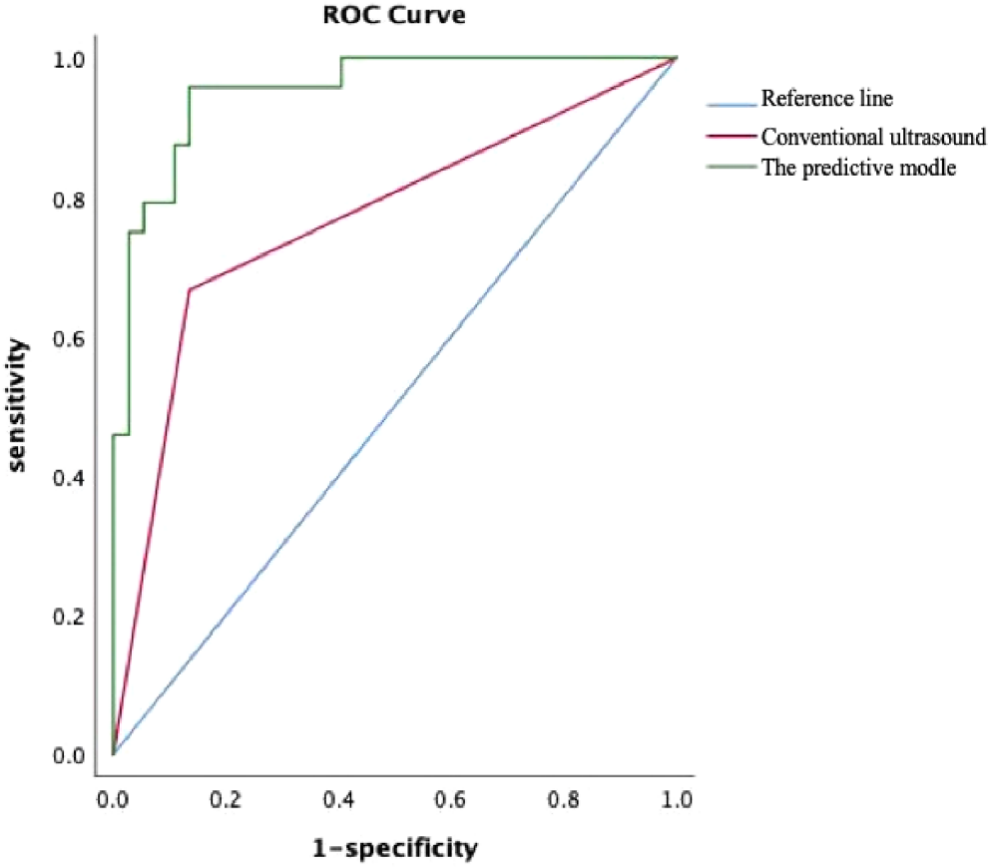
ROC curves of conventional ultrasound and the model's efficacy predicting lymph node metastasis

The ROC curve (Figure 5) was used to test conventional ultrasound and the model's predictive efficacy, the area under the curve was 0.76 (95% CI: 64%–89%, *p* = 0.000), 0.95 (95% CI: 90%–99%, *p* = 0.000), respectively. Hosmer–Lemeshow goodness of fit test showed X2 = 6.015, *p* = 0.65 (*p* > 0.05), indicating that the model had a high predictive value. The point nearest the upper left of the ROC curve corresponded to a sensitivity of 95% and specificity of 87%, the prediction probability (*p*) corresponding to this point was the cutoff, where *Y*(Logit(*P*)) was −0.80. When *Y* > −0.80, the model predicted that there would be metastasis, when *Y* < 0.80, the model predicted that there would be no metastasis.

## 4 DISCUSSION

ERUS has been recognized as the preferred imaging diagnostic method for patients with rectal cancer, and it is also one of the most accurate methods to evaluate the depth of rectal tumor invasion. Previous literature reported that the accuracy of ERUS in the evaluation of T stage of rectal cancer reached 86%,^11^ but the accuracy of ERUS in the evaluation of LN metastasis was low, only 77%.^12^ At present, there is still a lack of unified standard for the evaluation of LN metastasis by ultrasound.

According to previous research,^9^, ^13^, ^14^ lateral LNs with round, low echo and size of more than 5 mm are reliable basis for the differentiation of benign and malignant LNs. The results of this study showed that compared with the pathological results, the diagnostic efficiency was not high according to these conventional ultrasound features in diagnosing LN metastasis, the accuracy was only 79%, the specificity was 86%, the positive predictive value was 76%, the negative predictive value was 80%, and the sensitivity was only 67% in particular, in other words, about one‐third of the patients who actually had LN metastasis were missed as having no LN metastasis. Judging the shape of lateral LNs depends on the subjective judgment of the examiner. Although the size of LNs can be used as an objective indicator, the size of benign and malignant LNs has a large crossover range, and it is difficult to distinguish inflammatory reactive enlarged LNs from metastatic LNs only by this standard.

On the basis of previous research,^15^, ^16^, ^17^ in addition to incorporating the factor of conventional ultrasound features, this study also selected some other factors potentially affecting perirectal LN metastasis, then assigned values to these factors, and analyzed these factors by binary logistic regression method. Univariate analysis showed that circumferential angle of the tumor, UT stage, conventional ultrasound features diagnosis of LN metastasis, SR of tumor were risk factors for LN metastasis, while RI of rectal tumor was protective factor. Multivariate analysis showed that UT stage, conventional ultrasound features diagnosis of LN metastasis and SR were independent risk factors for LN metastasis. The regression model fitted by these variables had higher predictive value with 95% sensitivity and 87% specificity.

The results of this study showed that there was a close relationship between the depth of rectal tumor invasion and metastatic LNs, and the probability of LN metastasis was greater in patients with deeper invasion, and UT stage was an important risk factor for perirectal LN metastasis. Previous studies References 18, 19 had also pointed out that early rectal cancer had a lower risk of LN metastasis, which were confined to the mucosa because of its shallow infiltration depth. There was almost no lymphatic supply in the rectal mucosa. The intestinal lymphatic network was mainly located in the submucosa, while metastasis was more likely to occur when rectal tumors broke through the submucosa.

In addition, this study found that the stiffness of rectal tumors was also related to LN metastasis, and SR was higher in rectal tumors with LN metastasis than in tumors without LN metastasis, and the difference was statistically significant. Elastography reflects the stiffness of the lesion and has proven to be valuable in multiple organs such as the liver, breast, and prostate,^20^, ^21^, ^22^ malignant lesions have increased stiffness compared with normal tissue or benign lesions, and SR as a quantitative indicator improves examiner dependence. This study showed for every unit increase in SR, the risk of LN metastasis from rectal cancer increased approximately fivefold. Elastography is based on the B‐mode ERUS image, although it is an auxiliary means of B‐mode examination, to some extent it can compensate for the shortcomings of the B‐mode examination. When the intestinal wall is replaced by a large number of tumor cells and fibrous tissue, its biomechanics are altered. In particular, some of the larger tumors easily cause intestinal wall edema, vascular hyperplasia in the process of infiltration, which are not the infiltration of real tumor cells, the stiffness of these parts is lower than the real tumor tissue, which is difficult to be identified only by B‐mode image, while elastography helps to distinguish. Waage et al.^10^, ^23^, ^24^ also noted that SR differences between benign and malignant rectal tumors were statistically significant. The stiffness of advanced rectal cancer is greater than that of inchoate rectal cancer.

Limitations and prospects of this study: First, this model could not predict the LNs of T4 rectal cancer, which was a limitation of this study, but the most important significance of preoperative determination of LN metastasis was to choose the appropriate treatment plan, whether conservative treatment or radical surgery? As T4 rectal cancer was not suitable for radical surgery, the metastasis of LNs was not the most important for the choice of treatment direction. Second, as an important feature of tumor, the circumferential angle of the tumor showed no statistically significant difference in univariate analysis. Although RI of the tumor was different in the univariate analysis, it had no statistically significant effect on the results when entering the multivariate analysis. Such a result may be related to the insufficient sample size of this study. Third, it has been noted that the closer the LNs were to the primary tumor, the greater the chance of metastasis,^25^ and also the orientation of the tumor in the rectal may be an important influencing factor. These factors were not routinely observed in this study and therefore were not included in the analysis, and future studies will include these factors as well. Fourth, this study was retrospective, and there may be selective bias. The last, indocyanine green infrared imaging technology had shown preliminary advantages in the detection of sentinel LNs of rectal cancer,^26^ but there was still a lack of comparative studies with imaging. In the future, sentinel LN imaging screening for low rectal cancer metastasis is expected to be carried out through contrast‐enhanced ultrasound and ultrasound‐guided puncture.

## 5 CONCLUSION

Evaluation of lateral LN metastasis only based on conventional ERUS features of LNs was not effective enough. The three variables (UT stage of tumor, conventional ultrasound features diagnosis of LN metastasis, SR) were screened by logistic regression as independent risk factors for LN metastasis. After integrating these independent risk factors, the predictive model established greatly increased the diagnostic efficiency, comparisoning with that based on the morphology and size of LNs observed by conventional ultrasound alone. The predictive model (Logit(*P*) = −7.3 + 1.9 X10 + 2.1 X11 + 1.6 X13 (P: LN metastasis rate, X10: UT stage, X11: conventional ultrasound features diagnosis of LN metastasis, X13: SR) had good assessment efficacy and had certain clinical application value.

## CONFLICT OF INTEREST

The author has no conflict of interest to disclose.

## ETHICS STATEMENT

This study was approved by the Ethics Committee of the first affiliated hospital of Anhui University of Traditional Chinese Medicine (registration number: 2021MCZQ12). The requirement for individual consent was waived by the committee because of the retrospective nature of the study.

## Data Availability

The data that support the findings of this study are available on request from the corresponding author. The data are not publicly available due to privacy or ethical restrictions.
